# The Cross-Sectional Association Between Tinnitus and Actigraphy-Estimated Sleep in a Population-Based Cohort of Middle-Aged and Elderly Persons

**DOI:** 10.1097/AUD.0000000000001320

**Published:** 2022-12-23

**Authors:** Maud de Feijter, Berthe C. Oosterloo, André Goedegebure, Annemarie I. Luik

**Affiliations:** 1 Department of Epidemiology, Erasmus MC, University Medical Center, Rotterdam, the Netherlands; 2 Department of Otorhinolaryngology, Head and Neck Surgery, Erasmus MC, University Medical Center, Rotterdam, the Netherlands; 3 Department of Child and Adolescent Psychiatry/Psychology, Erasmus MC, University Medical Center, Rotterdam, the Netherlands.

**Keywords:** Actigraphy, Activity rhythms, Hearing loss, Population-based, Self-reported sleep, Tinnitus

## Abstract

Tinnitus is a common and burdensome disease, often accompanied by complaints of poor sleep. However, associations of tinnitus with objective estimates of sleep remain unclear, particularly in the general population. We assessed these associations in a population-based cohort of middle-aged and elderly persons.

**Design:**

This study included 1456 participants (mean age: 65.0 ± 7.1 years, 52% women) from the population-based Rotterdam Study. Tinnitus was self-reported and in those who reported tinnitus daily, symptom severity was assessed with the Tinnitus Handicap Inventory. We used actigraphy to estimate sleep and 24-hour activity rhythms objectively and sleep diaries to assess self-reported sleep. We estimated the difference in sleep and 24-hour activity rhythms first between those with and those without tinnitus and secondly with tinnitus severity.

**Results:**

Tinnitus, reported by 341 (23%) participants, and tinnitus severity, assessed in 194 participants with daily tinnitus, were not associated with actigraphy-estimated sleep or 24-hour activity rhythms, but were associated with a longer self-reported sleep onset latency (adjusted difference_tinnitus_ = 2.36, 95% confidence interval [CI] = 0.95–3.78, adjusted difference_tinnitus severity_ = 0.27, 95% CI = 0.013–0.54). After stratification for hearing loss, tinnitus was associated with longer self-reported sleep onset latency (adjusted difference = 2.26, 95% CI = 0.98–3.53) and less stable 24-hour activity rhythms (adjusted difference = −0.02, 95% CI = −0.04 to −0.00) in those with hearing loss. In those without hearing loss, tinnitus was associated with more stable rhythms (adjusted difference = 0.03, 95% CI = 0.01–0.05).

**Conclusions:**

Having tinnitus is associated with a longer self-reported sleep onset latency, but not with objective estimates of sleep, suggesting that the subjective experience of sleep may be particularly disturbed in those with tinnitus. In addition, hearing loss may modify the association of tinnitus and 24-hour activity rhythms.

## INTRODUCTION

Tinnitus is described as hearing a sound without an objective sound source being present (Han et al. 2021) and commonly occurs in the adult population (prevalence of 5–40%) (McCormack et al. 2016). For most people, tinnitus has no substantial impact on their life, but for 5 to 20% of those who experience tinnitus their functioning in daily life is significantly affected (McCormack et al. 2016).

Tinnitus has previously been associated with poor sleep in clinical (Salazar et al. 2019) and population-based samples (Wallhäusser-Franke et al. 2013; Stohler et al. 2019; Oosterloo et al. 2021). So far most studies assessing the association between tinnitus and sleep have focused on self-reported sleep quality or clinical diagnoses of insomnia, which are based on self-reported symptoms, and report more sleep complaints in those with tinnitus compared to healthy controls (Hébert & Carrier 2007; Langguth et al. 2011; Izuhara et al. 2013; Bhatt et al. 2016). In addition, several studies (Folmer & Griest 2000; Asnis et al. 2018; Lu et al. 2020; Inagaki et al. 2021; Li et al. 2022) reported the loudness and severity of tinnitus with the severity of sleep complaints. In a recent review by Teixeira and colleagues (Teixeira et al. 2018), only a few clinical studies assessed the association between tinnitus and sleep using objective estimates of sleep with conflicting findings. A prospective study by Hébert et al. (2011) reported more subjective sleep complaints in those with tinnitus compared to those without tinnitus, but no significant difference in polysomnography-measured sleep. Conversely, studies by Attanasio et al. (2013) and Burgos et al. (2005) reported significant differences in sleep architecture, such as more time spent in light sleep stages and a lower sleep efficiency, in those with chronic tinnitus compared to healthy controls. It is important to note that self-reported sleep mostly reflects the experience of sleep, whereas objectively estimated sleep may more reflect on the physiological aspect of sleep, potentially providing more insight into underlying biological mechanisms (Maglione et al. 2014; Mitrushina & Tomaszewski, 2019). As sleep is closely related to the 24-hour activity rhythm, a disturbed rhythm could potentially also explain the experience of poor sleep in those with tinnitus. Furthering our knowledge about these associations could help clinicians to improve treatment, reduce burden or even prevent development of sleep disturbances in those with tinnitus.

Tinnitus often co-occurs with hearing loss (Haider et al. 2018; Oosterloo et al. 2020), emphasizing the need to take hearing loss into account when studying the association of tinnitus and sleep. In addition, hearing loss is thought to be an accelerating factor for tinnitus severity (Haider et al. 2018; Oosterloo et al. 2020) and the origin of tinnitus between those with and without hearing loss may differ (Haider et al. 2018). So far, limited studies have however taken hearing loss into account when studying the association of tinnitus with sleep.

In this study, we therefore investigated the association of tinnitus and tinnitus severity with actigraphy-estimated sleep, self-reported sleep, and 24-hour activity rhythms in a population-based cohort of middle-aged and elderly persons. To gain insight in the effect of hearing loss on the association between tinnitus and sleep, we additionally stratified for hearing loss.

## MATERIALS AND METHODS

### Participants and Design

Within this study, we included participants from the Rotterdam Study, a population-based cohort of middle-aged and elderly inhabitants of Rotterdam, the Netherlands. This cohort was set up in 1990 with the main aim to examine neurological, cardiovascular, and other chronic age-related diseases. Details of the study design have been described by Ikram et al. (2020).

**Fig. 1.**
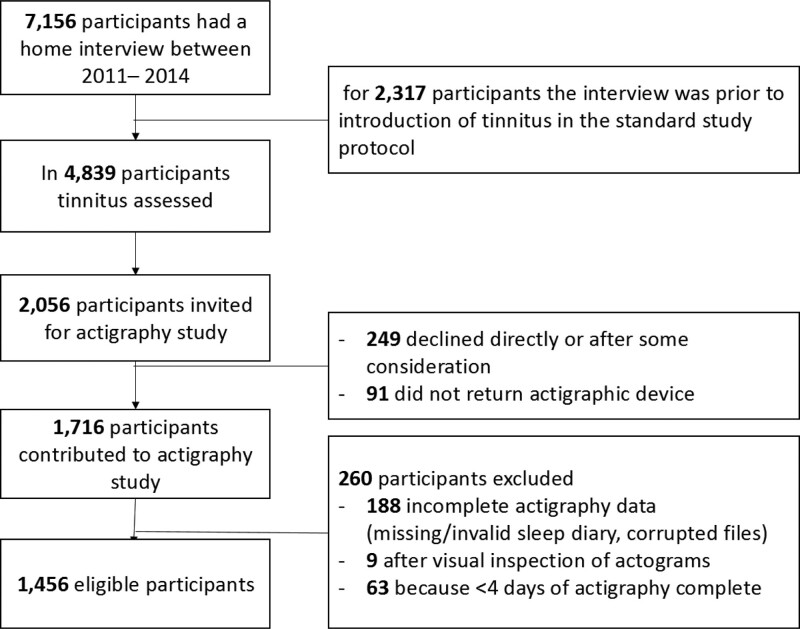
Flow diagram of the study population with actigraphy data available. Valid data for tinnitus and actigraphy was collected for 1456 participants between 2011 and 2014.

Between 2011 and 2014, tinnitus was assessed in 4839 participants during the home interview. Of those, 2056 participants were invited to participate in an actigraphy study, and 1807 agreed. We excluded those who did not return the actigraphy device or whose data was lost (n = 91), those with invalid actigraphy data (n = 197), and those with less than four complete days of actigraphy (n = 63), leaving 1456 eligible participants for analyses (Fig. 1).

The Rotterdam Study has been approved by the Medical Ethics Committee of the Erasmus MC (registration number MEC 02.1015) and by the Dutch Ministry of Health, Welfare and Sport (Population Screening Act WBO, license number 1071272-159521-PG). The Rotterdam Study has been entered into the Netherlands National Trial Register (http://www.trialregister.nl/trial/6645) meeting the requirements of the WHO International Clinical Trials Registry Platform (http://www.who.int/ictrp/network/primary-registries/) under shared catalog number NTR6831. All participants provided written informed consent to participate in the study and to have their information obtained from treating physicians. The authors assert that all procedures contributing to this work comply with the ethical standards of the relevant national and institutional committees on human experimentation and with the Helsinki Declaration of 1975, as revised in 2008.

### Assessment of Tinnitus

Tinnitus was assessed during the home interview. Participants were first asked whether they experienced sounds, such as whizzing, beeping, or humming, in (one of) their ears or in their head, without an objective external sound source present. Answer options to this question were: “no, never,” “yes, less than once a week,” “yes, more than once a week but not daily,” and “yes, daily.” If participants reported that tinnitus symptoms were present at least once a week, participants were considered to have tinnitus. Having symptoms less than once a week was not considered as tinnitus because of the heterogeneity and often temporary character of tinnitus complaints. When participants reported tinnitus symptoms at least daily, they were asked to complete the Dutch translation of a simplified Tinnitus Handicap Inventory (THI), a validated tool to estimate tinnitus severity in population-based settings (Baguley et al. 2000; Zeman et al. 2012). The THI contains 10 items and the total score ranges from 0 to 40, with a total score of ≥16 indicating a moderate to severe handicap (Newman et al. 2008).

### Assessment of Sleep

Participants were invited to wear an actigraph on their non-dominant wrist for 7 consecutive days and only remove it when bathing or during sauna visits. In addition, participants were asked to complete a daily sleep diary during the same week and to press a marker button on the actigraph at the time they wanted to initiate sleep (time to bed) and at the time they got out of bed (get-up time). We used the ActiWatch, model AW4 (Cambridge Technology Ltd, Cambridge, United Kingdom) or the GENEActiv (ActivInsight Ltd, Kimbolton United Kingdom). Recordings were sampled at 32 Hz (ActiWatch) or 50 Hz (GENEActiv) and scored per 30-second epoch, taking into account weighted scores of previous and following epochs. To distinguish sleep from wake a threshold of 20 was used (Kosmadopoulos et al. 2014). The *z* axis data of the tri-axial GENEActiv data were pre-analyzed to make records comparable with ActiWatch data (te Lindert & Van Someren 2013).

To estimate sleep, actigraphy data were combined with data from the sleep diary, using validated methods that have been previously described (te Lindert & Van Someren 2013), and averaged over the days with valid data available. If time to bed or get-up time was missing from the sleep diary, information from the actigraph marker button was used. Total sleep time (hours) was estimated as the total duration of epochs scored as sleep during the night. Sleep efficiency (%) was estimated as the proportion of time spent sleeping whilst lying in bed (100% × [total sleep time/time in bed]), with time in bed indicating the time between time to bed and get-up time. Sleep onset latency (minutes) was estimated as the duration between time to bed and sleep start. Lastly, wake after sleep onset (minutes) was defined as the total time scored as wake between sleep start and sleep end.

In addition, we estimated self-reported sleep using the sleep diaries. Total sleep time was estimated as the time participants estimated they had been sleeping that night. Sleep efficiency was the estimated time spent sleeping while lying in bed (100% × [total sleep time from the sleep diary/ time in bed]), where time in bed is calculated as explained earlier. Sleep onset latency was the estimated time it took to fall asleep. Lastly, wake after sleep onset was estimated as the time between self-reported sleep start and sleep end, minus the self-reported sleep duration. Each of these variables was averaged out overall nights the sleep diary was filled out.

### Assessment of 24-Hour Activity Rhythms

To assess 24-hour activity rhythms we estimated the interdaily stability (IS), intradaily variability, and onset of the least active 5 consecutive hours of the day (L5-onset) from the actigraphy recordings. Interdaily stability, indicating the stability of the sleep-wake rhythm over days, and intradaily variability, indicating fragmentation of the sleep-wake rhythm relative to its 24-hour amplitude were calculated using the nparACT R package on the actigraphy data (Blume et al. 2016). L5 onset was estimated as the average clock time on which the least active 5 consecutive hours of the day started (Van Someren, 1997).

### Other Variables

During the home interview, we obtained information on education, smoking, possible sleep apnea, and use of a hearing aid. Education was classified as primary education (primary), lower/intermediate general education or lower vocational education (low), intermediate vocational education or higher general education (middle), or higher vocational education or university (high) based on the UNESCO International Standard Classification of Education (United Nations Educational 1976). Smoking was classified as never, former, or current smoker. Possible sleep apnea was assessed as a binary variable using two questions from the Pittsburgh Sleep Quality Index (Buysse et al. 1991). Possible sleep apnea was considered present when participants reported (1) respiratory pauses during sleep at least 1 to 2 nights per week or (2) loud snoring at least 2 nights per week, combined with at least occasional respiratory pauses. Alcohol and coffee intake were assessed within the sleep diary. Alcohol intake indicated the number of days per week alcoholic beverages were consumed after 6 P.M. and coffee intake indicated the number of days per week coffee was consumed after 6 P.M. Use of a hearing aid was based on self-reported. Body mass index (BMI, kg/m^2^) and hearing loss were assessed at the research center. BMI was calculated by measuring height and weight on calibrated scales without heavy clothing and shoes. To estimate hearing loss, pure-tone audiometry data was collected by a trained healthcare professional in a soundproof booth. For both ears, air conduction thresholds were obtained for the frequencies 0.25, 0.5, 1, 2, 4, and 8 kHz (Ikram et al. 2020). We then determined pure-tone average hearing thresholds (PTA), averaged over 0.5, 1, 2, and 4 kHz, from the best hearing ear, as proposed by the WHO (World Health Organization, 2015). Hearing loss was defined as a PTA ≥25 decibel hearing level in the better ear (World Health Organization, 2015). Prevalent diabetes mellitus or cardiovascular disease were assessed based on data from medical record files. Lastly, the time in between the actigraphy sleep assessment and tinnitus was calculated in months.

### Statistical Analyses

Descriptive statistics were presented as number with percentage for categorical variables and mean with SD for numerical data. For our analyses, sleep and 24-hour activity rhythms variables were checked for extreme outliers (4 SD from the mean) and we set these to 4 SD from the mean in the same direction. Missing values for all covariates were less than 5% and handled by using multiple imputation. The MICE R package was used with five imputed datasets (Buuren & Groothuis-Oudshoorn 2010), for which we presented the pooled statistics (Rubin 2004). To correct for multiple testing, we used the false discovery rate to calculate the adjusted p-values, based on 10 determinants (Benjamini & Hochberg 1995).

First, cross-sectional linear regression models were used to estimate the association of tinnitus with actigraphy-estimated sleep, self-reported sleep, and 24-hour activity rhythms, comparing those with tinnitus to those without tinnitus. Second, to assess the effect of tinnitus severity on sleep and 24-hour activity rhythms, we used cross-sectional linear regression models to estimate the association of tinnitus severity, measured with the THI, with sleep and 24-hour activity rhythms in those experiencing tinnitus symptoms daily.

All associations were studied in a sex-age–adjusted model (model 1) and a model additionally adjusted for education, smoking behavior, intake of alcohol, intake of coffee, BMI, time between sleep and tinnitus assessment, and hearing loss (model 2). In addition, we estimated the associations by additionally adjusting for the use of a hearing aid (model 3) and after additionally adding prevalent diabetes mellitus or cardiovascular disease (model 4).

To test the effect of hearing loss on the association of tinnitus with sleep, we repeated all analyses stratified for hearing loss. Furthermore, for comparison purposes, we also estimated the association of hearing loss with sleep and 24-hour activity rhythms. Lastly, because the association between tinnitus and sleep has been reported to differ between men and women (Gu et al. 2021; Richter et al. 2021), we reran our analyses stratified for sex.

Analyses were performed in R version R 3.5.3 (R Foundation for Statistical Computing, Vienna, Austria, www.R-project.org).

## RESULTS

We included 1456 participants (mean age of 65.0 ± 7.1 years, 52% women; Table 1). In total, 341 (23.4%) participants reported the experience of tinnitus (Table 1). In the 194 participants for whom we assessed tinnitus severity with the THI the median score was 4 (IQR 0–10) and 30 participants had clinically relevant symptoms (THI ≥16). Hearing loss was present in 664 (45.6%) of the participants, of whom 198 reported to experience tinnitus.

**TABLE 1.** Baseline characteristics of the study population at baseline (N = 1456)

	N	(%)	Mean/Median	SD/IQR
Demographics				
Age (yrs)			65.03	7.13
Women	755	51.9		
Education				
Primary	103	7.1		
Low	531	36.5		
Intermediate	425	29.2		
High	390	26.8		
Health indicators				
Smoking				
Non smoker	475	32.6		
Former smoker	809	55.6		
Current smoker	170	11.7		
Alcohol (units/d)			2.73	2.62
Coffee (units/d)			4.45	2.89
Body mass index (kg/m^2^)			27.62	4.26
Hearing threshold (dB HL)			26.80	13.07
Hearing loss*	664	45.6		
Tinnitus				
Tinnitus	341	23.4		
Tinnitus severity (score)†‡			4	0–10
Actigraphy-estimated sleep				
Total sleep time (h:min)			6 h:14 min	56 min
Sleep efficiency (%)			75.94	8.36
Sleep onset latency (min)			19 min	17 min
Wake after sleep onset (min)			57 min	27 min
Self-reported sleep				
Total sleep time (h:min)			6 h:49 min	56 min
Sleep efficiency (%)			82.37	10.93
Sleep onset latency (min)			18 min	11 min
Wake after sleep onset (min)			68 min	82 min
24-h activity rhythms				
Interdaily stability (score)			0.74	0.12
Intradaily variability (score)			0.46	0.15
L5 onset (24-h clock time)			1:16	1:19
Time between measurements of sleep and tinnitus (mos)‡			3	2–5

For categorical variables, the absolute number (%) is indicated, and for numeric variables the mean ± SD. Education was missing for seven participants (0.4%), smoking for 2 (0.1%), alcohol intake for 2 (0.1%), coffee intake for 2 (0.1%), body mass index for 1 (0.1%), and hearing loss for 128 (8.8%). For other variables, there are no missing values.

* Hearing loss was defined as an average hearing level of ≥25 dB in the better ear.

† Assessed using the Tinnitus Handicap Inventory (n = 194).

‡ Median and interquartile range were presented.

L5 onset, onset of the least active 5 consecutive hours of the day.

Tinnitus and tinnitus severity were not associated with actigraphy-estimated sleep (Table 2). When sleep was self-reported, we observed that tinnitus was associated with a longer sleep onset latency (adjusted difference per minute = 2.36, 95% confidence interval [CI] = 0.95–3.78; Table 3). In addition, we observed an association of tinnitus with lower self-reported sleep efficiency (adjusted difference per minute = −1.42, 95% CI = −2.82 to −0.02), but this association did not hold after multiple testing corrections. Results did not change substantially after additional correction for the use of a hearing aid or after additional correction for prevalent diabetes mellitus or cardiovascular disease (data not shown). No associations of tinnitus and tinnitus severity with 24-hour activity rhythms were observed (Table 4).

**TABLE 2.** The cross-sectional association of tinnitus compared to no tinnitus and of tinnitus severity with actigraphy-estimated sleep

	Total Sleep Time (min)		Sleep Efficiency (%)		Sleep Onset Latency (min)		Wake After Sleep Onset (min)	
	Adjusted Difference (95% CI)	*p*	Adjusted Difference (95% CI)	*p*	Adjusted Difference (95% CI)	*p*	Adjusted Difference (95% CI)	*p*
Tinnitus (N = 1456)								
Model 1	0.05 (−0.06 to 0.16)	0.39	−0.07 (−1.09 to 0.95)	0.90	0.60 (−1.44 to 2.65)	0.56	−0.88 (−4.07 to 2.31)	0.59
Model 2	0.04 (−0.08 to 0.16)	0.54	−0.02 (−1.12 to 1.07)	0.97	0.22 (−1.97 to 2.41)	0.84	−1.74 (−5.14 to 1.67)	0.32
Tinnitus severity (N = 194)								
Model 1	0.01 (−0.01 to 0.03)	0.30	0.01 (−0.15 to 0.18)	0.87	0.07 (−0.26 to 0.40)	0.68	0.02 (−0.43 to 0.47)	0.93
Model 2	0.01 (−0.01 to 0.03)	0.34	0.02 (−0.16 to 0.20)	0.82	0.00 (−0.35 to 0.35)	1.00	0.11 (−0.35 to 0.56)	0.65

Effect estimates were obtained using cross-sectional linear regression models, adjusted for sex, and age (model 1), and adjusted for sex, age, education, smoking, alcohol intake, coffee intake, body mass index, time between sleep and tinnitus assessment, and hearing loss (model 2).

CI, confidence interval.

**TABLE 3.** The cross-sectional association of tinnitus compared to no tinnitus and of tinnitus severity with self-reported sleep

	Total Sleep Time (min)		Sleep Efficiency (%)		Sleep Onset Latency (min)		Wake After Sleep Onset (min)	
	Adjusted Difference (95% CI)	*p*	Adjusted Difference (95% CI)	*p*	Adjusted Difference (95% CI)	*p*	Adjusted Difference (95% CI)	*p*
Tinnitus (N = 1456)								
Model 1	−0.08 (−0.19 to 0.03)	0.16	−1.13 (−2.433 to 0.183)	0.09	2.48 (1.15 to 3.81)	<0.001*	−0.29 (−11.66 to 11.09)	0.96
Model 2	−0.11 (−0.23 to 0.01)	0.08	−1.42 (−2.82 to −0.02)	0.047	2.36 (0.95 to 3.78)	0.001*	−0.83 (−13.34 to 11.67)	0.90
Tinnitus severity (N = 194)								
Model 1	0.00 (−0.02 to 0.02)	0.73	−0.17 (−0.39 to 0.06)	0.15	0.27 (0.02 to 0.51)	0.032	1.61 (0.06 to 3.17)	0.043
Model 2	0.00 (−0.02 to 0.02)	0.79	−0.16 (−0.40 to 0.08)	0.19	0.27 (0.01 to 0.54)	0.040	1.58 (−0.15 to 3.32)	0.073

Effect estimates were obtained using cross-sectional linear regression models, adjusted for sex, and age (model 1), and adjusted for sex, age, education, smoking, alcohol intake, coffee intake, body mass index, time between sleep and tinnitus assessment, and hearing loss (model 2).

* *P*-value remained significant (<0.05) after correcting for multiple testing, using the false discovery rate.

CI, confidence interval.

**TABLE 4.** The cross-sectional association of tinnitus compared to no tinnitus and of tinnitus severity with actigraphy-estimated 24-hour activity rhythms

	Intradaily Stability (Score)		Interdaily Variability (Score)		L5 Onset (h)	
	Adjusted Difference (95% CI)	*p*	Adjusted Difference (95% CI)	*p*	Adjusted Difference (95% CI)	*p*
Tinnitus (N = 1456)						
Model 1	−0.01 (−0.02 to 0.01)	0.45	0.01 (−0.01 to 0.02)	0.53	0.09 (−0.07 to 0.25)	0.29
Model 2	0.00 (−0.02 to 0.02)	0.95	0.00 (−0.02 to 0.02)	0.92	0.08 (−0.09 to 0.24)	0.38
Tinnitus severity (N = 194)						
Model 1	0.00 (0.00 to 0.00)	0.12	0.00 (0.00 to 0.00)	0.33	0.01 (−0.02 to 0.04)	0.47
Model 2	0.00 (0.00 to 0.00)	0.32	0.00 (0.00 to 0.00)	0.63	0.01 (−0.02 to 0.04)	0.37

Effect estimates were obtained using cross-sectional linear regression models, adjusted for sex, and age (model 1), and adjusted for sex, age, education, smoking, alcohol intake, coffee intake, body mass index, time between sleep and tinnitus assessment, and hearing loss (model 2).

CI, confidence interval; L5 onset, onset of the least active 5 consecutive hours of the day.

After stratification for hearing loss, again no associations between tinnitus and actigraphy-estimated sleep were observed in either group (Supplemental Table 1, Supplemental Digital Content 1, http://links.lww.com/EANDH/B80). In those with hearing loss, we did observe an association of tinnitus with long self-reported sleep onset latency (adjusted difference = 2.80, 95% CI = 0.91–4.70) and short self-reported total sleep time (adjusted difference = −0.19, 95% CI = −0.04 to 0.00, not significant after multiple testing correction) and of tinnitus severity with long self-reported wake after sleep onset (adjusted difference = 2.26, 95% CI = 0.98–3.53; Supplemental Table 2, Supplemental Digital Content 1, http://links.lww.com/EANDH/B80). In those without hearing loss, no associations with self-reported sleep were found. Lastly, in those with hearing loss, tinnitus was associated with a lower stability of 24-hour activity rhythms (adjusted difference = −0.02, 95% CI = −0.04 to 0.00), but in those without hearing loss tinnitus was associated with a higher stability (adjusted difference = 0.03, 95% CI = 0.01–0.05) after adjustment for confounders (Supplemental Table 3, Supplemental Digital Content 1, http://links.lww.com/EANDH/B80). These associations did not remain significant after multiple testing corrections. For comparison purposes, we also assessed the association of hearing loss with sleep and 24-hour activity rhythms, no significant associations were observed (Supplemental Tables 4–6, Supplemental Digital Content 1, http://links.lww.com/EANDH/B80).

Finally, stratification for sex showed some differences between men and women. In men, we observed an association of tinnitus with low stability of the 24-hour activity rhythm (adjusted difference = −0.02, 95% CI = −0.04 to 0.00), whereas in women tinnitus was associated with a high stability of the rhythm (adjusted difference = 0.02, 95% CI = 0.00–0.05). Moreover, we only found associations of tinnitus with self-reported sleep in men (self-reported total sleep time: adjusted difference = −0.22, 95% CI = −0.38 to −0.06; self-reported sleep onset latency: adjusted difference = 3.22, 95% CI = 1.33–5.11). In contrast, in women, an association of tinnitus with actigraphy-estimated sleep was found (actigraphy-estimated total sleep time: adjusted difference = 0.20, 95% CI = 0.03–0.37). Tinnitus severity associated with long self-rated sleep onset latency (adjusted difference = 0.43, 95% CI = 0.07–0.80) and long self-rated wake after sleep onset (adjusted difference = 0.02, 95% CI = 0.01–0.03) only in men (Supplemental Tables 1–3, Supplemental Digital Content 1, http://links.lww.com/EANDH/B80).

## DISCUSSION

Our results suggest that tinnitus and tinnitus severity are not associated with objective estimates of sleep and 24-hour activity rhythms. However, when sleep was self-reported, we did observe an association of having tinnitus and more severe tinnitus with a longer sleep onset latency. After stratification for hearing loss, we observed an association of tinnitus with lower stability of 24-hour activity rhythms, shorter total sleep time, and longer self-reported sleep onset latency in those with hearing loss, but with a higher stability in those without hearing loss. In addition, tinnitus severity was associated with longer self-reported wake after sleep onset in those with hearing loss, but not in those without hearing loss.

We did not observe any associations of tinnitus or tinnitus severity with actigraphy-estimated sleep and 24-hour activity rhythms, suggesting that tinnitus is not associated with objective disturbances of sleep and 24-hour activity rhythms. These findings might seem to contradict previous literature which indicates that objectively estimated sleep disturbances are more common in patients with a clinical diagnosis of tinnitus than in healthy controls (Teixeira et al. 2018; Stohler et al. 2019). Yet, the severity of the tinnitus in these patients was likely substantially higher than in our cohort as these were patients who have actively sought treatment for their tinnitus. In contrast, our study used a population-based approach with no pre-selection on tinnitus occurrence, severity, or actively seeking care. The absent association could also be explained by a habituation to the tinnitus including lowering of associated distress (Simões et al. 2021). Potentially, distress is most prominent shortly after the onset of tinnitus. Over time, people may then learn to cope with the constant noise, reducing the associated hyperarousal (Simões et al. 2021), and consequently reducing sleep problems. Within our study, we unfortunately were not able to test this effect of time as we do not have information on the duration of the complaints. However, if distress and hyperarousal are lower, we would expect that this also leads to less self-reported sleep complaints, unless these subjective complaints become the focus point of the distress and hyperarousal. The sleep complaints are then unlikely to improve without targeted sleep treatment (Pyrke et al. 2017).

Our results indeed suggest that there is an association of tinnitus with longer self-reported sleep onset latency. Where previous studies have mainly assessed sleep retrospectively (Bhatt et al. 2016; Stohler et al. 2019; Oosterloo et al. 2021), we demonstrate that this association remains when using a prospective sleep diary. Our results therefore indicate that this association cannot merely be explained by the poorer recollection of sleep in those with tinnitus. Longer self-reported sleep onset latency in those with tinnitus could potentially be explained by increased awareness of sleep, or worry about a lack thereof, in those with tinnitus (Riemann et al. 2010; Musiek et al. 2018; Leng et al. 2019; Mitrushina & Tomaszewski 2019). In addition, participants might feel it takes a long time to fall asleep, because the experience of a tinnitus-related sound is unpleasant and increases awareness. Together with previous work, our results support that the experience of sleep is particularly affected in those with tinnitus, even when the tinnitus can be classified as subclinical in the majority of cases. Therefore, daily life might be affected even more than recognized in those with tinnitus.

We observed substantial differences in the association of tinnitus with sleep and 24-hour activity rhythms after stratification for hearing loss. Overall those with hearing loss and tinnitus had more self-reported sleep complaints than those without hearing loss and tinnitus, although the association was opposite for the stability of the 24-hour activity rhythm. Important to note is that although those with hearing loss reported daily tinnitus more often they did not report a higher tinnitus severity and that hearing loss was not associated with any sleep variables. As hearing loss was also not associated with any of the sleep estimates per se, the associations of tinnitus with sleep cannot be explained by hearing loss. This supports that hearing loss may be an effect modifier in the association between tinnitus and sleep, in other words, tinnitus may impact sleep differently in those with and without hearing loss, but is not more influential than tinnitus for sleep. This maybe explained because tinnitus pathophysiology accompanied by hearing loss is thought to be caused more often by hearing-related factors, such as hearing damage caused by an external source (Haider et al. 2018). Furthermore, in those suffering from tinnitus and hearing loss, both disorders might have been induced by frequent exposure to noise at least for a subgroup of these participants (Henry et al. 2020). We could speculate that jobs with frequent exposure to loud noise, such as in the nightlife and entertainment sector, might also be associated with poorer self-reported sleep and less stable 24-hour activity rhythms, even after retirement. In the absence of hearing loss, tinnitus pathophysiology is more likely to be explained by alterations in brain regions involved in hearing (Haider et al. 2018). This underlying neuropathology (Langguth et al. 2013), may lead to complaints that people try to compensate by strongly regulating their daily routine, resulting in stable 24-hour activity rhythms (de Feijter et al. 2020). Potentially, tinnitus-related sleep disturbances require more targeted interventions that take into account hearing loss.

Lastly, we observed that tinnitus was associated with self-reported sleep in men, whereas tinnitus was associated with actigraphy-estimated sleep in women. In addition, tinnitus was associated with low stability in men, but with high stability in women. As these results are similar to those after stratification for hearing loss, a likely explanation might be that tinnitus is more frequently accompanied by hearing loss in men. Within our study, we were not able to further investigate potential underlying mechanisms explaining the observed differences between men and women, which is why we recommend further research into potential sex-specific associations.

Several limitations need to be taken into account when interpreting these results. First, we were not able to draw conclusions on temporality or causality due to a lack of sufficient repeated measurements. Second, our measurements for tinnitus were based on self-report and not confirmed by a clinician. Third, no information was available on the duration of tinnitus occurrence, therefore we are not able to this take into account. Fourth, the tinnitus burden in our population-based cohort is relatively low. Therefore, potential associations might have been missed with relatively little variation. Last, actigraphy was collected only in a subsample of participants as an additional measurement. This might have introduced some level of selection bias as those willing to participate in the actigraphy study were more healthy and higher educated, as opposed to those who refused participation. Nevertheless, studying the association between tinnitus and objective sleep estimates in a large population-based sample is unique in the field and allowed us to provide new insights into the association between tinnitus and sleep.

In conclusion, within a population of middle-aged and elderly persons we demonstrated an association of tinnitus and tinnitus severity with longer self-reported sleep onset latency, but not with actigraphy-estimated measures for sleep or 24-hour activity rhythms. This suggests that the subjective experience of sleep may be particularly disturbed in those with tinnitus.

## ACKNOWLEDGMENTS

The Rotterdam Study is funded by Erasmus Medical Center and Erasmus University, Rotterdam, the Netherlands Organization for the Health Research and Development (ZonMw), the Research Institute for Diseases in the Elderly (RIDE), the Ministry of Education, Culture and Science, the Ministry of Health, Welfare and Sports, the European Commission (DG XII), and the Municipality of Rotterdam.

## Supplementary Material
