# Supplementary material for: The Cross-Sectional Association Between Tinnitus and Actigraphy-Estimated Sleep in a Population-Based Cohort of Middle-Aged and Elderly Persons
Source: Ear Hear. 2022 Dec 23;44(4):732–9. doi: 10.1097/AUD.0000000000001320 (PMC10262987; doi:10.1097/AUD.0000000000001320)
Supplement: Supplementary file 1 [file aud-44-732-s001.pdf]

SUPPLEMENT

Supplementary Table 1. The cross-sectional association of tinnitus compared to no tinnitus and of tinnitus severity with actigraphy-estimated sleep, stratified for hearing loss and stratified for sex.

	Total sleep time (min)		Sleep efficiency (%)		Sleep onset latency (min)		Wake after sleep onset (min)	
	**Adjusted difference (95%CI)**	**p-value**	**Adjusted difference (95%CI)**	**p-value**	**Adjusted difference (95%CI)**	**p-value**	**Adjusted difference (95%CI)**	**p-value**
Hearing loss								
Tinnitus (N=664)	0.03 (-0.13;0.187)	0.73	0.11 (-1.38;1.61)	0.88	1.54 (-1.50;4.59)	0.32	-3.46 (-8.17;1.25)	0.15
Tinnitus severity (N=120)	0.00 (-0.02;0.02)	0.82	-0.13 (-0.36;0.10)	0.26	0.11 (-0.38;0.60)	0.65	0.50 (-0.12;1.11)	0.11
No hearing loss								
Tinnitus (N=664)	0.04 (-0.14;0.23)	0.66	-0.20 (-1.85;1.46)	0.82	-1.81 (-5.01;1.39)	0.27	0.36 (-4.64;5.36)	0.89
Tinnitus severity (N=55)	0.03 (-0.01;0.06)	0.12	0.24 (-0.01;0.49)	0.06	-0.29 (-0.68;0.10)	0.14	-0.44 (-1.03;0.14)	0.14
Men								
Tinnitus (N=701)	-0.11 (-0.27;0.06)	0.20	-0.52 (-2.20;1.15)	0.54	1.82 (-1.39;5.03)	0.27	-1.56 (-6.95;3.82)	0.57
Tinnitus severity (N=108)	0.01 (-0.02;0.03)	0.58	-0.03 (-0.30;0.23)	0.80	0.16 (-0.41;0.72)	0.58	0.09 (-0.57;0.76)	0.78
Women								
Tinnitus (N=755)	0.20 (0.03;0.37)	0.024	0.50 (-0.92;1.93)	0.49	-1.76 (-4.76;1.25)	0.25	-1.61 (-5.80;2.59)	0.45
Tinnitus severity (N=86)	0.01 (-0.02;0.04)	0.48	0.08 (-0.15;0.32)	0.48	-0.19 (-0.57;0.19)	0.33	0.28 (-0.36;0.92)	0.39

Abbreviations: CI, Confidence Interval; Effect estimates were obtained using cross-sectional linear regression models, adjusted for sex, age, education, smoking, alcohol intake, coffee intake, body mass index, time between sleep and tinnitus assessment, and hearing loss (Model 2).

Supplementary Table 2. The cross-sectional association of tinnitus compared to no tinnitus and of tinnitus severity with self-reported sleep, stratified for hearing loss and stratified for sex.

	Total sleep time (min)		Sleep efficiency (%)		Sleep onset latency (min)		Wake after sleep onset (min)	
	**Adjusted difference (95%CI)**	**p-value**	**Adjusted difference (95%CI)**	**p-value**	**Adjusted difference (95%CI)**	**p-value**	**Adjusted difference (95%CI)**	**p-value**
<u>Hearing loss</u>								
Tinnitus (N=664)	-0.19 (-0.35;-0.02)	0.026	-1.70 (-3.57;0.18)	0.08	2.80 (0.91;4.70)	0.004*	-3.95 (-18.40;10.50)	0.59
Tinnitus severity (N=120)	0.00 (-0.03;0.02)	0.79	-0.25 (-0.53;0.03)	0.07	0.23 (-0.12;0.58)	0.19	2.26 (0.98;3.53)	0.001*
<u>No hearing loss</u>								
Tinnitus (N=664)	-0.02 (-0.20;0.17)	0.87	-1.11 (-3.26;1.04)	0.31	1.79 (-0.37;3.95)	0.10	5.35 (-17.30;27.99)	0.64
Tinnitus severity (N=55)	-0.01 s(-0.04;0.02)	0.51	-0.13 (-0.56;0.29)	0.53	0.35 (-0.06;0.75)	0.09	2.19 (-2.91;7.29)	0.39
<u>Men</u>								
Tinnitus (N=701)	-0.22 (-0.38;0.06)	0.008	-1.59 (-3.53;0.34)	0.11	3.22 (1.33;5.11)	0.001	0.66 (-15.94;14.61)	0.93
Tinnitus severity (N=108)	-0.01 (-0.03;0.02)	0.60	-0.26 (-0.56;0.05)	0.10	0.43 (0.07;0.80)	0.021	0.02 (0.01;0.03)	0.011
<u>Women</u>								
Tinnitus (N=755)	0.02 (-0.17;0.20)	0.86	-1.16 (-3.22;0.90)	0.27	-1.58 (-0.43;3.69)	0.14	-0.58 (-21.20;19.52)	0.94
Tinnitus severity (N=86)	-0.01 (-0.04;0.03)	0.73	-0.13 (-0.50;0.24)	0.49	0.08 (-0.32;0.48)	0.71	0.01 (-0.02;0.05)	0.49

Abbreviations: CI, Confidence Interval; Effect estimates were obtained using cross-sectional linear regression models, adjusted for sex, age, education, smoking, alcohol intake, coffee intake, body mass index, time between sleep and tinnitus assessment, and hearing loss (Model 2). * P-value remained significant (<0.05) after correcting for multiple testing, using FDR.

Supplementary Table 3. The cross-sectional association of tinnitus compared to no tinnitus and of tinnitus severity with actigraphy-estimated 24-hour activity rhythms, stratified for hearing loss and stratified for sex.

	Intradaily stability (score)		Interdaily variability (score)		L5 onset (hour)	
	**Adjusted difference (95%CI)**	**p-value**	**Adjusted difference (95%CI)**	**p-value**	**Adjusted difference (95%CI)**	**p-value**
Hearing loss						
Tinnitus (N=664)	-0.02 (-0.04;0.00)	0.038	0.01 (-0.02;0.04)	0.42	0.08 (-0.15;0.31)	0.51
Tinnitus severity (N=120)	0.00 (-0.01;0.00)	0.27	0.00 (0.00;0.00)	0.93	0.01 (-0.03;0.05)	0.63
No hearing loss						
Tinnitus (N=664)	0.03 (0.01;0.05)	0.011	-0.01 (-0.04;0.02)	0.37	0.07 (-0.18;0.32)	0.59
Tinnitus severity (N=55)	0.00 (-0.00;0.00)	0.92	0.00 (0.00;0.01)	0.38	0.04 (-0.01;0.08)	0.11
Men						
Tinnitus (N=701)	-0.02 (-0.04;0.00)	0.040	0.01 (-0.02;0.04)	0.47	0.55 (-0.75;1.85)	0.40
Tinnitus severity (N=108)	0.00 (-0.01;0.00)	0.21	0.00 (-0.01;0.00)	0.67	0.10 (-0.12;0.32)	0.37
Women						
Tinnitus (N=755)	0.02 (0.00;0.05)	0.047	-0.01 (-0.03;0.02)	0.58	-0.74 (-2.27;0.79)	0.34
Tinnitus severity (N=86)	0.00 (0.00;0.00)	0.83	0.00 (0.00;0.01)	0.58	-0.16 (-0.42;0.11)	0.25

Abbreviations: L5 onset, onset of the least active 5 consecutive hours of the day; CI, Confidence Interval; Effect estimates were obtained using cross-sectional linear regression models, adjusted for sex, age, education, smoking, alcohol intake, coffee intake, body mass index, time between sleep and tinnitus assessment, and hearing loss (Model 2). None of the p-values remained significant (<0.05) after correcting for multiple testing, using FDR.

Supplementary Table 4. The cross-sectional association of hearing loss compared to no hearing loss with actigraphy-estimated sleep

	Total sleep time (min)		Sleep efficiency (%)		Sleep onset latency (min)		Wake after sleep onset (min)	
	**Adjusted difference (95%CI)**	**p-value**	**Adjusted difference (95%CI)**	**p-value**	**Adjusted difference (95%CI)**	**p-value**	**Adjusted difference (95%CI)**	**p-value**
Hearing loss (N=1,328)								
Model 1	0.00 (-0.12;0.11)	0.98	0.21 (-0.84;1.25)	0.70	-0.41 (-2.50;1.69)	0.70	2.93 (-0.31;6.18)	0.08
Model 2	0.01 (-0.11;0.12)	0.89	0.36 (-0.70;1.42)	0.50	-0.99 (-3.10;1.12)	0.36	2.28 (-1.00;5.56)	0.17

Abbreviations: CI, Confidence Interval; Effect estimates were obtained using cross-sectional linear regression models, adjusted for sex, and age (Model 1), and adjusted for sex, age, education, smoking, alcohol intake, coffee intake, body mass index, time between sleep and tinnitus assessment, and hearing loss (Model 2).

Supplementary Table 5. The cross-sectional association of hearing loss compared to no hearing loss with self-reported sleep.

	Total sleep time (min)		Sleep efficiency (%)		Sleep onset latency (min)		Wake after sleep onset (min)	
	**Adjusted difference (95%CI)**	**p-value**	**Adjusted difference (95%CI)**	**p-value**	**Adjusted difference (95%CI)**	**p-value**	**Adjusted difference (95%CI)**	**p-value**
Hearing loss (N=1,328)								
Model 1	0.06 (-0.06;0.17)	0.33	1.09 (-0.25;2.43)	0.11	0.21 (-1.14;1.56)	0.76	-4.77 (-16.85;7.32)	0.44
Model 2	0.03 (-0.09;0.15)	0.58	0.90 (-0.45;2.25)	0.19	0.31 (-1.06;1.67)	0.66	-4.57 (-16.84;7.70)	0.47

Abbreviations: CI, Confidence Interval; Effect estimates were obtained using cross-sectional linear regression models, adjusted for sex, and age (Model 1), and adjusted for sex, age, education, smoking, alcohol intake, coffee intake, body mass index, time between sleep and tinnitus assessment, and hearing loss (Model 2).

Supplementary Table 6. The cross-sectional association of hearing loss compared to no hearing loss with actigraphy-estimated 24-hour activity rhythms.

	Intradaily stability (score)		Interdaily variability (score)		L5 onset (hour)	
	**Adjusted difference (95%CI)**	**p-value**	**Adjusted difference (95%CI)**	**p-value**	**Adjusted difference (95%CI)**	**p-value**
Hearing loss (N=1,328)						
Model 1	0.00 (-0.01;0.02)	0.60	-0.01 (-0.02;0.01)	0.62	0.12 (-0.04;0.28)	0.15
Model 2	0.01 (-0.01;0.02)	0.34	-0.01 (-0.03;0.01)	0.31	0.09 (-0.07;0.25)	0.27

Abbreviations: L5 onset, onset of the least active 5 consecutive hours of the day; CI, Confidence Interval; Effect estimates were obtained using cross-sectional linear regression models, adjusted for sex, and age (Model 1), and adjusted for sex, age, education, smoking, alcohol intake, coffee intake, body mass index, time between sleep and tinnitus assessment, and hearing loss (Model 2).
